# Training quality, laboratory skills readiness, and employability of medical laboratory graduates in post-conflict Somalia: a Structural Equation Modeling (SEM) study

**DOI:** 10.1186/s12909-026-09682-x

**Published:** 2026-06-15

**Authors:** Abdirahman Ibrahim Abdi, Anas Ali Alhur, Ahmed Mohamed Yusuf, Mohamed Osman Abdi Idris

**Affiliations:** 1 Faculty of Education, SIMAD University, Mogadishu, Somalia; 2 Department of Health Informatics, College of Public Health and Health Informatics, University of Hail, Hail, Saudi Arabia; 3 College of Public Health, Imam Abdulrahman Bin Faisal University, Dammam, Saudi Arabia

**Keywords:** Medical laboratory education, Graduate employability, Student self-efficacy, Job readiness, Structural equation modeling

## Abstract

Medical laboratory workforce readiness is critical for strengthening health systems in fragile and post-conflict settings. However, empirical evidence on how educational quality translates into employability remains limited. This study examined the associations between curriculum relevance, practical training exposure, and instructor competency on employment outcomes among medical laboratory students and graduates in Mogadishu, Somalia, with student self-efficacy and job readiness modeled as mediating mechanisms. Using a quantitative cross-sectional design, data were collected from 291 final-year students and recent graduates and analyzed using Partial Least Squares Structural Equation Modeling. The measurement model demonstrated satisfactory reliability and validity. The structural results indicated that curriculum relevance, practical training exposure, and instructor competency significantly predicted student self-efficacy, whereas curriculum relevance and self-efficacy significantly predicted job readiness. Student self-efficacy also had a significant positive effect on employment outcomes, whereas job readiness did not exhibit a direct effect. Mediation analysis further revealed that student self-efficacy significantly mediated the relationship between practical training exposure and both job readiness and employment outcomes, while job readiness did not serve as a significant mediator, and sequential mediation pathways were not supported. The model explained 68.3% of the variance in student self-efficacy and 58.2% in job readiness, but only 2.3% in employment outcomes, indicating limited predictive power for the final outcome variable. These findings highlight the central role of psychological readiness mechanisms in understanding the relationship between training quality and employability in post-conflict health education systems. The study contributes theoretically by testing a multistage mediation framework grounded in Social Cognitive and Human Capital theories and offers policy-relevant insights for curriculum reform, experiential learning enhancement, and faculty development to strengthen health workforce preparedness.

## Introduction

Medical laboratory services are a critical component of effective healthcare systems, supporting accurate diagnosis, disease surveillance, and evidence-based clinical decision making. Globally, the quality of health outcomes is closely linked to the competence and preparedness of the laboratory workforce, making training quality and skills development central to strengthening health systems [5, 15]. The World Health Organization has emphasized that well-trained laboratory professionals are essential for achieving universal health coverage and improving patient safety, particularly in low- and middle-income countries, where diagnostic capacity remains limited [31, 32]. Prior research in health professions education indicates that curriculum relevance, adequate practical training, and instructor competency are among the most important determinants of graduates’ professional confidence and readiness to enter the workforce [13]. Unlike medical or nursing education, medical laboratory training is highly technical and diagnostic-focused, requiring precision in analytical procedures, strict adherence to quality standards, and extensive hands-on laboratory practice, making the alignment between training and workplace demands particularly critical in this field.

In sub-Saharan Africa, medical laboratory education continues to face substantial challenges related to inadequate training infrastructure, limited access to modern diagnostic equipment, shortages of qualified instructors, and weak partnerships between universities and clinical training sites [5, 15, 31, 32]. These constraints often result in graduates entering the labor market with insufficient hands-on experience and limited confidence in performing routine lab procedures. Specifically, graduates may lack practical competencies in areas such as specimen handling, microbiological techniques, quality assurance procedures, and the use of modern laboratory equipment, as well as essential soft skills, including communication, teamwork, and professional confidence in clinical environments**.** Although several studies have examined general health workforce shortages and training gaps in the region, far fewer have empirically investigated how specific educational quality factors translate into employability outcomes through psychological and professional readiness mechanisms, particularly using advanced analytical approaches capable of modeling complex structural relationships.

This situation is further compounded in post-conflict contexts, such as Somalia, where prolonged instability has disrupted health system development and weakened educational infrastructure. Despite the recent expansion of medical and health sciences programs in Somali universities, healthcare facilities continue to report shortages of skilled laboratory personnel, and employers frequently express concerns about graduate preparedness for clinical laboratory environments. Limitations in clinical placement opportunities, inconsistent supervision, and resource constraints may hinder students’ development of practical competencies and professional confidence. Specifically, employers reported gaps in practical diagnostic competencies, such as specimen handling, microbiological techniques, quality assurance procedures, and the use of modern laboratory equipment, as well as soft skills, including communication, teamwork, and professional confidence in clinical environments.

However, systematic empirical evidence on how training conditions affect graduate employability in the Somali context remains extremely limited, and no known studies have examined these relationships using Structural Equation Modeling.

Existing literature on graduate employability in health education has largely relied on descriptive statistics or traditional regression models, which are limited in their ability to simultaneously assess the direct and indirect relationships among multiple interrelated factors. Moreover, the mediating roles of student self-efficacy and job readiness in linking educational quality to employment outcomes remain underexplored, especially in fragile and post-conflict settings such as Somalia. Understanding these mediating mechanisms is essential, as self-efficacy influences individuals’ confidence in performing professional tasks, and job readiness reflects the practical and behavioral preparedness required for successful workplace integration. Recent employability research also emphasizes that graduate employment is shaped by the alignment between universities, industry expectations, graduate skills, and workplace performance requirements. In post-conflict environments such as Somalia, the application of these theoretical perspectives is shaped by structural constraints, including limited training infrastructure, resource shortages, and unstable labor market conditions, which may affect the association between educational quality, psychological readiness, and employment outcomes [13, 31, 32]. The absence of integrated structural models addressing these pathways represents both a theoretical and methodological gap in the current literature.

Accordingly, this study aims to examine how curriculum relevance, practical training exposure, and instructor competency are associated with employment outcomes of medical laboratory graduates in post-conflict Somalia, both directly and indirectly through student self-efficacy and job readiness, using Structural Equation Modeling. By testing a model that links training quality factors to psychological confidence, professional preparedness, and employment outcomes, the study seeks to provide a more comprehensive understanding of the graduate transition process from education to labor market participation in fragile health systems.

This study makes several important contributions. Theoretically, it integrates educational quality constructs with employability and psychological readiness variables within a single analytical framework, extending employability research to the field of medical-laboratory education. Methodologically, SEM was applied to simultaneously test multiple direct and mediated pathways, allowing a more comprehensive examination of the relationships among variables rather than implying causality. Contextually, it provides the first known empirical evidence on the determinants of laboratory graduate employability in post-conflict Somalia, a setting largely absent from the global health workforce literature. From a policy and practice perspective, the findings are expected to inform curriculum reform, clinical training partnerships, and faculty development strategies aimed at improving workforce readiness and strengthening diagnostic services in fragile healthcare systems.

### Literature review

#### Theoretical framework

This study is grounded in Social Cognitive Theory (SCT) and Social Cognitive Career Theory (SCCT) which explain how learning environments shape career-related outcomes through psychological mechanisms, specifically the self-efficacy. According to SCT, individuals’ beliefs about their capabilities influence their motivation, persistence, and performance in work-related tasks [2]. In learning settings, self-efficacy can be developed through mastery experiences, instructor feedback, and direct practice. SCCT also describes the effects of self-efficacy on career behaviors and outcomes, including the perceptions of individuals about readiness, expectations of outcomes and the participation in career related behaviors, including job searching and adjustment at work [24]. Economically and in terms of skills, Human Capital Theory asserts that the investment in the relevant education and training enhances productivity and employability, on the condition that relevant skills gained in the labor market meet the demands [4]. Moreover, graduate employability models also focus on the fact that employment results are not determined by mere academic knowledge but also by confidence, transferable skills, and readiness to work [8, 33]. Collectively, these theories support a model in which training quality is indirectly linked to employment outcomes via self-efficacy and job readiness.

Beyond applying established theories, the present study advances the employability literature by integrating educational quality factors, psychological readiness mechanisms, professional readiness, and employment outcomes within a single multistage mediation framework. While prior employability studies have often examined curriculum quality, self-efficacy, job readiness, or employment outcomes separately, this study simultaneously investigates how curriculum relevance, practical training exposure, and instructor competency are associated with employability through student self-efficacy and job readiness. The model further contributes conceptually by testing direct, indirect, and sequential mediation pathways, thereby showing whether educational quality is linked to employment outcomes directly or through psychological confidence and professional preparedness. Furthermore, by applying these pathways to post-conflict Somalia, the study extends SCT, SCCT, and Human Capital Theory to a context characterized by institutional constraints, workforce shortages, limited clinical training infrastructure, and labor market instability. This provides a more context-sensitive understanding of how educational quality translates into employability in fragile health systems.

#### Curriculum relevance and student outcomes

Curriculum relevance can be defined as the extent to which academic learning correlates with job activity, professional requirements, and emerging industry requirements. When students feel that the curriculum as taught to them is directly relevant to their actual job requirements, they tend to build confidence in their work skills and consider themselves ready to work [33]. In health professions education, empirical research has demonstrated that education based on applied competencies and problem-solving in clinical situations has a positive impact on self-efficacy and perception of job readiness [8, 12]. Nevertheless, in resource-constrained and post-conflict environments, curricula do not quickly keep pace with technological and service delivery shifts, which may diminish the education-employment connection. Recent studies have similarly reported that alignment between curriculum content, workplace competencies, industry expectations, and skill development remains a key determinant of graduate employability and workforce preparedness in higher education settings [14, 21, 29]. Although relevant, curriculum relevance has seldom been tested as an explicit latent variable in SEM models of graduate employability in weak health systems, which is a significant knowledge gap.

#### Practical training exposure and skills development

Exposure to practical training is generally known to be a key factor in professional competence and employability in health education. Experiential learning theory underlines that the best way to acquire skills is to make learners active in real tasks and reflect on what they experience in the real world [1, 6, 23, 26]. Clinical placements, laboratory, and simulation-based training help students use theoretical knowledge, hone technical skills, and acquire professional behaviors. According to some studies, increased exposure to practical experience leads to better confidence, competence, and perceived clinical work readiness [1, 6, 10, 25, 26]. Nonetheless, in most Sub-Saharan African and post-conflict locations, training institutions are deficient, the quality of placements is very diversified, and it remains unclear whether practical exposure always warrants employability benefits [3]. Recent evidence further suggests that experiential learning and workplace-based training contribute significantly to professional confidence, employability skills, and workforce readiness among health-related graduates [13, 20, 21, 29]. SEM-based studies rarely examine how exposure to practice can interact with curriculum and instructors’ factors with the effect of psychological mediators, and that is what this study aims to achieve.

#### Instructor competency and learning effectiveness

Instructor competency is a key factor that influences the learning process, confidence, and professional identification of students. Skilled teachers do not just pass technical information; they also provide constructive feedback, demonstrate professional behavior, and assist students in handling complicated processes in the laboratory. Social Cognitive Theory states that self-efficacy development can and should be contributed by verbal persuasion and social modeling by credible mentors [2]. Medical and laboratory education empirical studies indicate that instructor experience and quality of teaching have a strong impact on the perceived competence and readiness of students to practice in a professional environment [12]. However, although prior research has examined instructor effects, it often does not account for their interaction with the relevance of the curriculum and realistic exposure in the formation of employability results. Another gap in the literature is this absence of built-in modeling.

#### Student self-efficacy and job readiness

Self-efficacy shows the assurance that students have in their capability to accomplish professional duties and their capability to deal with difficulties at work. SCCT suggests that self-efficacy affects how people would assess their level of employment preparedness and engage in the career-seeking process [24]. Students with higher self-efficacy tend to be involved in skill development, adjust to new work situations, and persevere in job searches [2]. Job readiness, on the other hand, demonstrates the practical, behavioral, and attitudinal readiness to be effectively employed in the workplace in terms of communication skills, professionalism, and flexibility [8]. Although self-efficacy and job readiness are closely related, they represent different dimensions of employability. Self-efficacy reflects students’ confidence in their ability to perform professional tasks, whereas job readiness reflects their perceived preparedness to enter and function in the workplace. Therefore, self-efficacy is treated in this study as a psychological mechanism that may strengthen job readiness, rather than as the same construct. Nevertheless, few studies have modelled this linear connection with SEM, especially in health professions education studies.

#### Job readiness and employment outcomes

Job readiness is commonly viewed as an important predictor of employment outcomes, including successful hiring, faster job acquisition, and early job performance. Most employers usually prefer graduates who have practical competence, teamwork skills, and professional behavior over those with pure academic results [33]. Job-ready laboratory graduates are particularly important in health systems because they can be integrated into service delivery with minimal additional training, which is essential in resource-constrained environments**.** Despite numerous studies that recognize the significance of readiness, none have empirically examined its mediating role between training quality and employment outcomes, particularly in post-conflict settings where employment opportunities can be mediated by the instability of institutions and labor market absorption capacity. Recent research has also emphasized that graduate employability is influenced not only by individual competencies and readiness but also by broader labor market conditions, institutional capacity, and workforce absorption mechanisms [5, 15, 19, 31, 32]. Thus, job readiness may improve graduates’ preparedness for work, but it may not always translate directly into employment outcomes when labor market absorption, institutional hiring systems, and health-sector vacancies are limited.

In general, existing literature supports the relevance of curriculum alignment, practical training exposure, and instructor competency in shaping graduate preparedness. However, findings vary across contexts and disciplines, and many studies rely on descriptive or regression-based approaches rather than integrated structural models. In Sub-Saharan African and fragile health systems, employability is shaped not only by graduate competence but also by limited clinical training infrastructure, weak institutional capacity, restricted health-sector job absorption, and unstable hiring systems [5, 15, 19, 31, 32]. These conditions may weaken the direct translation of educational quality and job readiness into actual employment outcomes, even when graduates report confidence and professional preparedness. Medical laboratory education also remains underrepresented in employability research compared with nursing and medical education.

Building on the above theoretical perspectives, this study conceptualizes student self-efficacy and job readiness as key mediating mechanisms linking training quality to employment outcomes. According to Social Cognitive Career Theory, educational experiences influence career-related outcomes indirectly through psychological processes such as self-efficacy, which shape confidence, motivation, and behavioral readiness [24]. In this context, training quality factors, including curriculum relevance, practical training exposure, and instructor competency, are expected to enhance student self-efficacy, which may subsequently improve job readiness and employment outcomes. Job readiness represents a proximal outcome of self-efficacy and reflects the practical, behavioral, and professional preparedness required for labor market entry [8]. Accordingly, the proposed pathway suggests that training quality may strengthen student self-efficacy, which then enhances job readiness and contributes to employment-related outcomes. This framework provides a context-sensitive explanation of how educational quality may translate into employability in post-conflict health systems, where structural constraints can weaken direct relationships and make psychological readiness pathways more important.

##### Hypotheses

**Fig. 1:**
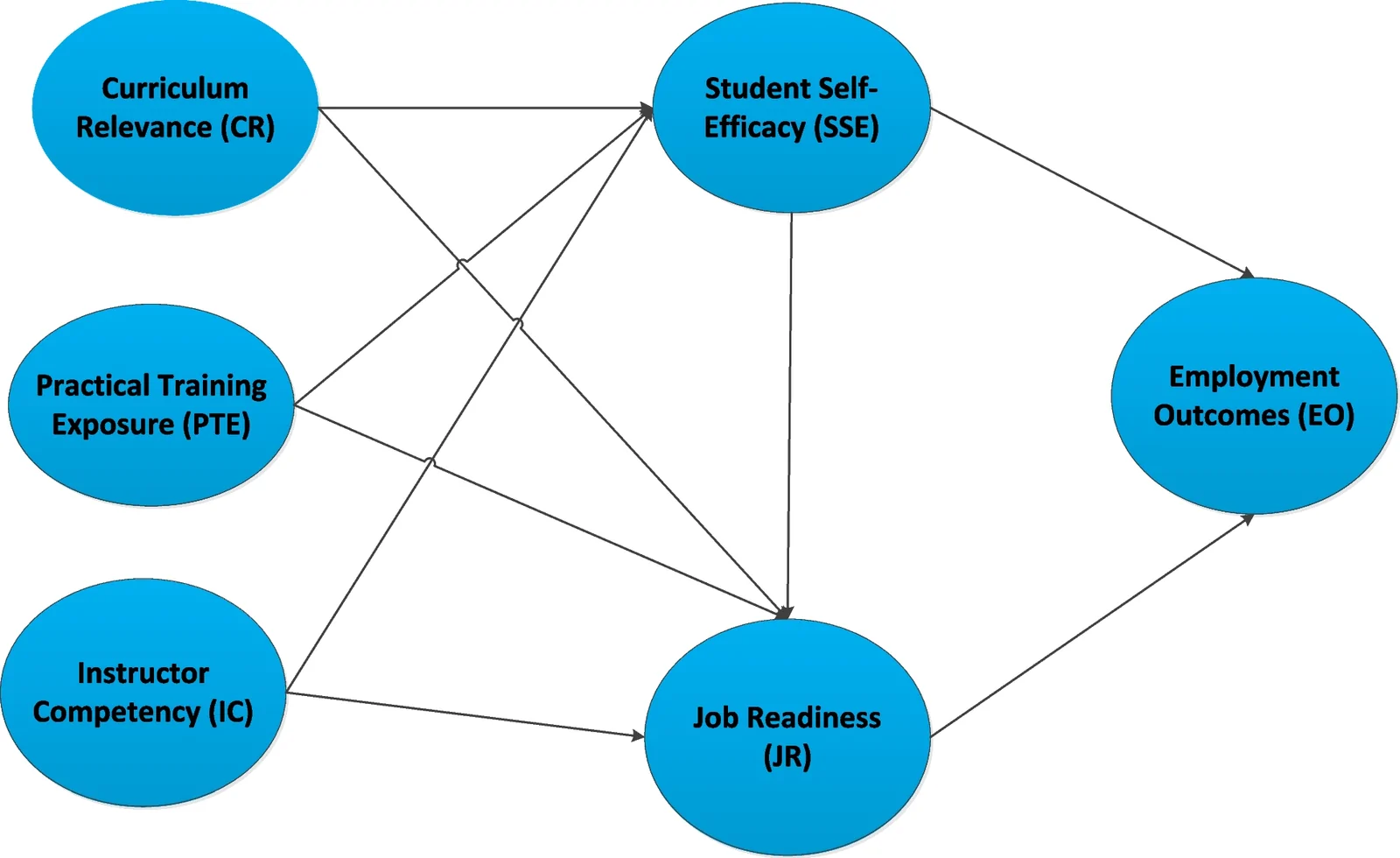
Conceptual framework of training quality, self-efficacy, job readiness, and employment outcomes among medical laboratory graduates

The hypotheses of the study, based on the conceptual framework in Fig. 1, propose that curriculum relevance, practical training exposure, and instructor competency are exogenous constructs associated with student self-efficacy and job readiness, which, in turn, are associated with employment outcomes. Drawing on Social Cognitive Theory, Social Cognitive Career Theory, Human Capital Theory, and graduate employability models, the following hypotheses were formulated:

- Direct Effects Hypotheses
  - H1: Curriculum relevance (CR) has a positive effect on student self-efficacy (SSE).
  - H2: Practical training exposure (PTE) positively affects student self-efficacy (SSE).
  - H3: Instructor competency (IC) positively affects student self-efficacy (SSE).
  - H4: Curriculum relevance (CR) positively affects job readiness (JR).
  - H5: Practical training exposure (PTE) positively affects job readiness (JR).
  - H6: Instructor competency (IC) positively affects job readiness (JR).
  - H7: Student self-efficacy (SSE) positively affects job readiness (JR).
  - H8: Job readiness (JR) positively affects employment outcomes (EO).
  - H9: Student self-efficacy (SSE) positively affects employment outcomes (EO).
- Indirect (Mediation) Hypotheses
  (A) Mediation via Student Self-Efficacy (SSE)
    H10: Student self-efficacy (SSE) mediates the relationship between curriculum relevance (CR) and job readiness (JR).
    H11: Student self-efficacy (SSE) mediates the relationship between practical training exposure (PTE) and job readiness (JR).
    H12: Student self-efficacy (SSE) mediates the relationship between instructor competency (IC) and job readiness (JR).
    H13: Student self-efficacy (SSE) mediates the relationship between curriculum relevance (CR) and employment outcomes (EO).
    H14: Student self-efficacy (SSE) mediates the relationship between practical training exposure (PTE) and employment outcomes (EO).
    H15: Student self-efficacy (SSE) mediates the relationship between instructor competency (IC) and employment outcomes (EO).
  (B) Mediation via Job Readiness (JR)
    H16: Job readiness (JR) mediates the relationship between curriculum relevance (CR) and employment outcomes (EO).
    H17: Job readiness (JR) mediates the relationship between practical training exposure (PTE) and employment outcomes (EO).
    H18: Job readiness (JR) mediates the relationship between instructor competency (IC) and employment outcomes (EO).
    H19: Job readiness (JR) mediates the relationship between student self-efficacy (SSE) and employment outcomes (EO).
  (C) Sequential Mediation (SSE → JR → EO)
    H20: Student self-efficacy (SSE) and job readiness (JR) sequentially mediate the relationship between curriculum relevance (CR) and employment outcomes (EO).
    H21: Student self-efficacy (SSE) and job readiness (JR) sequentially mediate the relationship between instructor competency (IC) and employment outcomes (EO).
    H22: Student self-efficacy (SSE) and job readiness (JR) sequentially mediate the relationship between practical training exposure (PTE) and employment outcomes (EO).

## Methodology

### Research design

This study adopted a quantitative, cross-sectional, correlational design to examine the relationships between training quality factors, psychological readiness, and employment outcomes among medical laboratory graduates in post-conflict Somalia. A correlational design is appropriate when the objective is to assess theoretically grounded relationships among latent constructs without experimental manipulation, particularly within a Structural Equation Modeling (SEM) framework. Based on Social Cognitive Career Theory and graduate employability models, this study focused on how curriculum relevance, practical training exposure, and instructor competency are associated with employment outcomes indirectly through student self-efficacy and job readiness.

### Participants

A purposive sampling technique was used to select participants based on the predefined inclusion criteria. A non-probability sampling approach is appropriate when the target population possesses specific educational and professional characteristics relevant to the research objectives [9]. The sample comprised final-year medical laboratory students and recent graduates from accredited universities in Mogadishu who had completed both academic coursework and clinical laboratory training.

A total of 291 participants were included in the final analysis, comprising 62.5% final-year students and 37.5% recent graduates. The sample was predominantly male (68.4%), which reflects the enrollment patterns in the study context but may introduce limitations for generalizability. This gender distribution is consistent with broader trends in health and science education participation in the region; however, it may introduce potential bias and limit the generalizability of the findings, particularly regarding female graduates’ experiences and employability outcomes. Therefore, the results should be interpreted with caution when extending the conclusions across gender groups. Academic status was not incorporated as a control variable in the PLS-SEM model. Instead, it was reported as an important demographic and inclusion-relevant participant characteristic because the sample included both final-year students and recent graduates. This distinction may influence experience, exposure to the labor market, and readiness levels, and therefore should be considered when interpreting the findings. Future studies may include academic status as a formal control variable by adding direct paths to key endogenous constructs such as job readiness and employment outcomes.

The sample size met the recommended thresholds for Partial Least Squares Structural Equation Modeling (PLS-SEM), ensuring adequate statistical power for estimating complex mediation models [17].

### Instruments

Data were collected using a structured self-report questionnaire developed by adapting validated instruments from prior studies in health professions education, employability research, and Social Cognitive Career Theory. The questionnaire measured six latent constructs: curriculum relevance, practical training exposure, instructor competency, student self-efficacy, job readiness, and employment outcomes.

Specifically, student self-efficacy items were informed by Social Cognitive Theory-based scales [2], job readiness items were adapted from graduate employability frameworks [8], and employment outcomes were operationalized as perceived employability and alignment with current work roles. Each construct was measured using five reflective items, resulting in a total of 30 measurement items across all constructs (Table 2).

The adaptation process involved the systematic modification of existing validated scales to ensure contextual relevance to the Somali medical laboratory education environment. This process was conducted by the research team in collaboration with subject-matter experts and included rewording items to reflect laboratory-specific tasks, aligning terminology with local clinical practice, and simplifying the language to ensure clarity and respondent comprehension.

Content validity was established through an expert review involving three specialists in medical laboratory education and two experts in health professions training and curriculum development. These experts evaluated the instrument for clarity, relevance, and representativeness of each construct, and minor revisions were made based on their feedback.

The revised instrument was subsequently pilot-tested with a small sample of 20 medical laboratory graduates to assess its clarity, reliability, and contextual appropriateness. Feedback from the pilot test led to minor wording adjustments to improve item clarity and to ensure cultural suitability.

Curriculum relevance assesses the alignment between academic content and professional laboratory practice. Practical training exposure captured the adequacy of clinical placements and hands-on diagnostic experience. Instructor competency reflects perceived teaching effectiveness, supervision quality, and feedback. Student self-efficacy measures confidence in performing laboratory and professional tasks. Job readiness assesses technical, behavioral, and professional preparedness for employment. Employment outcomes were operationalized as perceived employability, current employment status (e.g., employed, internship, or job-seeking), and the degree of alignment between acquired skills and current or expected job role. In this study, employment outcomes were treated as a broad employability-related construct rather than a purely objective employment-status measure. This operationalization was used because many respondents were final-year students, interns, volunteers, or recent graduates who were still transitioning from education to formal employment. Therefore, the construct captures both perceived employability and early workforce-position indicators. However, this broader operationalization may introduce conceptual ambiguity and should be interpreted with caution. Accordingly, the employment outcomes construct reflects a combination of subjective and objective employability indicators. The subjective component relates to graduates’ perceptions of their employability and preparedness for employment, whereas the objective component includes current employment status and the alignment between acquired competencies and work roles. This broader operationalization was considered appropriate because actual labor market entry among participants was still evolving, making a purely objective measure of employability insufficient for capturing early career outcomes in the Somali post-conflict context.

All items were rated on a five-point Likert scale ranging from strongly disagree to strongly agree.

### Data collection

Data were collected through supervised, in-person questionnaire administration over six weeks. Research assistants were trained to explain the study objectives, procedures, and ethical considerations. Participation was voluntary, and the respondents were informed of their right to withdraw at any time without consequences.

No personally identifiable information was collected, and confidentiality and anonymity were strictly enforced. Face-to-face data collection was employed due to the limited digital infrastructure and to enhance response quality in a post-conflict context. The immediate collection of completed questionnaires helped minimize missing data and non-response bias.

### Data analysis

Partial Least Squares Structural Equation Modeling (PLS-SEM) using SmartPLS 4 was employed for data analysis. PLS-SEM was used throughout the analysis, and the abbreviation was consistently applied after its first definition. The choice of PLS-SEM over covariance-based SEM (CB-SEM) was guided by several methodological considerations that aligned with the study’s objectives and data characteristics. First, the study examines a relatively complex structural model involving multiple exogenous constructs and mediating relationships (i.e., student self-efficacy and job readiness), for which PLS-SEM is particularly suitable because of its ability to efficiently estimate complex models and indirect effects.

Second, the primary objective of the study is to explain and predict key endogenous constructs—specifically, student self-efficacy, job readiness, and employment outcomes—rather than to achieve strict theory confirmation. In this regard, PLS-SEM is appropriate because it focuses on maximizing the explained variance (R^2^) and predictive accuracy.

Third, PLS-SEM is a variance-based approach that does not require multivariate normality and performs well with moderate sample sizes. Given the sample size (*N* = 291) and the use of Likert-scale survey data, which may deviate from a normal distribution, PLS-SEM provides a more flexible and reliable estimation approach.

Although this study is grounded in established theoretical frameworks (e.g., Social Cognitive Theory and Human Capital Theory), it applies these theories within a relatively under-researched, context-specific setting (post-conflict Somalia). Therefore, an analytical approach that supports both theory extension and prediction is appropriate for this study. In contrast, the CB-SEM is more suitable for strict model confirmation under conditions of large samples and normally distributed data.

Accordingly, PLS-SEM is considered the most appropriate analytical technique for this study [17]. This methodological choice is further supported by recent methodological guidance and empirical applications showing that PLS-SEM is suitable for prediction-oriented models, complex structural relationships, and mediation analysis in applied educational and social science research [17, 28, 30]. These studies provide empirical support for the appropriateness of PLS-SEM in analyzing multidimensional educational constructs in applied research contexts.

The analysis followed a two-step approach: the assessment of the measurement model, followed by the evaluation of the structural model. Specifically, the analytical procedure was conducted in a systematic sequence to ensure its robustness and transparency. First, data screening was performed to check for missing values, outliers and response consistency. Mean replacement was applied for minimal missing data, and no severe outliers were detected in the data.

Indicator reliability was assessed using outer loadings, with values above 0.70 being considered ideal. Loadings between 0.60 and 0.70 were retained if supported by composite reliability and theoretical relevance. Internal consistency reliability was evaluated using Composite Reliability (acceptable range: 0.70–0.95). Convergent validity was assessed using the Average Variance Extracted (AVE > 0.50). Discriminant validity was assessed using the heterotrait–monotrait (HTMT) ratio of correlations, applying both a conservative threshold of 0.85 and a more liberal threshold of 0.90 [17, 18]. In addition, discriminant validity was further assessed using the Fornell–Larcker criterion, whereby the square root of the Average Variance Extracted (AVE) for each construct was compared with its correlations with other constructs. Discriminant validity is established when the square root of the AVE exceeds the inter-construct correlations, indicating that each construct shares more variance with its indicators than with other constructs [11]. Furthermore, cross-loadings were examined to ensure that each indicator loaded highest on its associated construct compared to all other constructs, thereby confirming indicator-level discriminant validity and minimizing potential construct overlap.

Collinearity was assessed using the Variance Inflation Factor (VIF), with values below 3.3 indicating the absence of multicollinearity and a reduced likelihood of common method bias [22]. To further strengthen the assessment of common method bias, Harman’s single-factor test was conducted, and the results indicated that no single factor accounted for the majority of the variance, suggesting that common method bias is unlikely to significantly affect the results. In addition, the use of full collinearity VIF values below the recommended threshold provides further support that common method variance is not a serious concern in this study.

Structural relationships were evaluated using standardized path coefficients (β), t-values, and *p*-values obtained through bootstrapping with 5,000 resampling. In addition, 95% bias-corrected confidence intervals were examined to assess the robustness and statistical significance of estimated relationships. The model explanatory power was assessed using coefficients of determination (R^2^), with values of 0.75, 0.50, and 0.25 indicating substantial, moderate, and weak explanatory power, respectively [17]. Effect sizes (f^2^) were interpreted as small (0.02), medium (0.15), and large (0.35) [7]. Predictive relevance was assessed using the Stone–Geisser Q^2^ statistic obtained through blindfolding, where values greater than zero indicated the predictive relevance of the model.

Finally, mediation analysis was conducted by examining the indirect effects using bootstrapping procedures. The significance of the mediation effects was determined based on bootstrapped indirect effects and their corresponding confidence intervals, following the recommended procedures for mediation testing in SEM [27].

## Results

### Demographic characteristics of respondents

Table 1 presents the demographic characteristics and inclusion-relevant participant characteristics of the study respondents (*N* = 291). The sample was predominantly male (68.4%, *n* = 199), while females constituted 31.6% (*n* = 92) of the sample. With respect to age distribution, the largest proportion of participants were aged over 30 years (41.6%, *n* = 121), followed by those aged ≤ 20 years (27.1%, *n* = 79), 26–30 years (17.5%, *n* = 51), and 21–25 years (13.7%, *n* = 40) (Table 1).

**Table 1:** Demographic characteristics and inclusion-relevant participant characteristics of respondents

Variable	Category	Frequency (n)	Percentage (%)
Gender	Male	199	68.4
	Female	92	31.6
	Total	291	100.0
Age Group (years)	≤ 20	79	27.1
	21–25	40	13.7
	26–30	51	17.5
	> 30	121	41.6
	Total	291	100.0
Academic Status	Final-year students	182	62.5
	Recent graduates	109	37.5
	Total	291	100.0
Employment Status	Employed	89	30.6
	Unemployed/Seeking	53	18.2
	Internship/Volunteer	149	51.2
	Total	291	100.0
Years Since Graduation	< 1 year	83	28.5
	1–2 years	26	8.9
	> 2 years	87	29.9
	Still students	95	32.6
	Total	291	100.0

In terms of academic status, 62.5% (*n* = 182) of the respondents were final-year students, whereas 37.5% (*n* = 109) were recent graduates. Regarding employment status, more than half of the participants were engaged in internship or volunteer positions (51.2%, *n* = 149), reflecting a transitional entry into the health workforce, while 30.6% (*n* = 89) were employed and 18.2% (*n* = 53) were unemployed or actively seeking employment (Table 1).

Regarding post-graduation experience, 32.6% (*n* = 95) of respondents were still students, 29.9% (*n* = 87) reported more than two years since graduation, 28.5% (*n* = 83) had graduated within the past year, and 8.9% (*n* = 26) had one to two years since graduation. All participants met the predefined inclusion criteria, including the completion of both academic coursework and supervised clinical laboratory training components, ensuring eligibility for analysis (Table 1).

The demographic composition reflects a heterogeneous cohort of students and early career graduates actively transitioning from education to the labor market, which directly aligns with the study’s objective of examining how training quality factors influence student self-efficacy, job readiness and employment outcomes. The high proportion of interns, volunteers, and recent graduates provides a relevant empirical basis for testing mediation pathways within the Structural Equation Modeling framework, particularly in a post-conflict health system where workforce integration and employment trajectories remain fluid. This diversity strengthens the contextual validity and interpretability of the structural relationships in the model.

The observed gender imbalance should be considered when interpreting the findings, as it may influence the generalizability of the structural relationships between male and female participants.

### Measurement model

The measurement model was evaluated to establish the reliability and validity of the latent constructs, curriculum relevance, practical training exposure, instructor competency, student self-efficacy, job readiness, and employment outcomes, prior to assessing the structural relationships. Following established guidelines for Partial Least Squares Structural Equation Modeling (PLS-SEM), the assessment included indicator reliability, internal consistency reliability, convergent validity, and discriminant validity [16, 17].

**Fig. 2:**
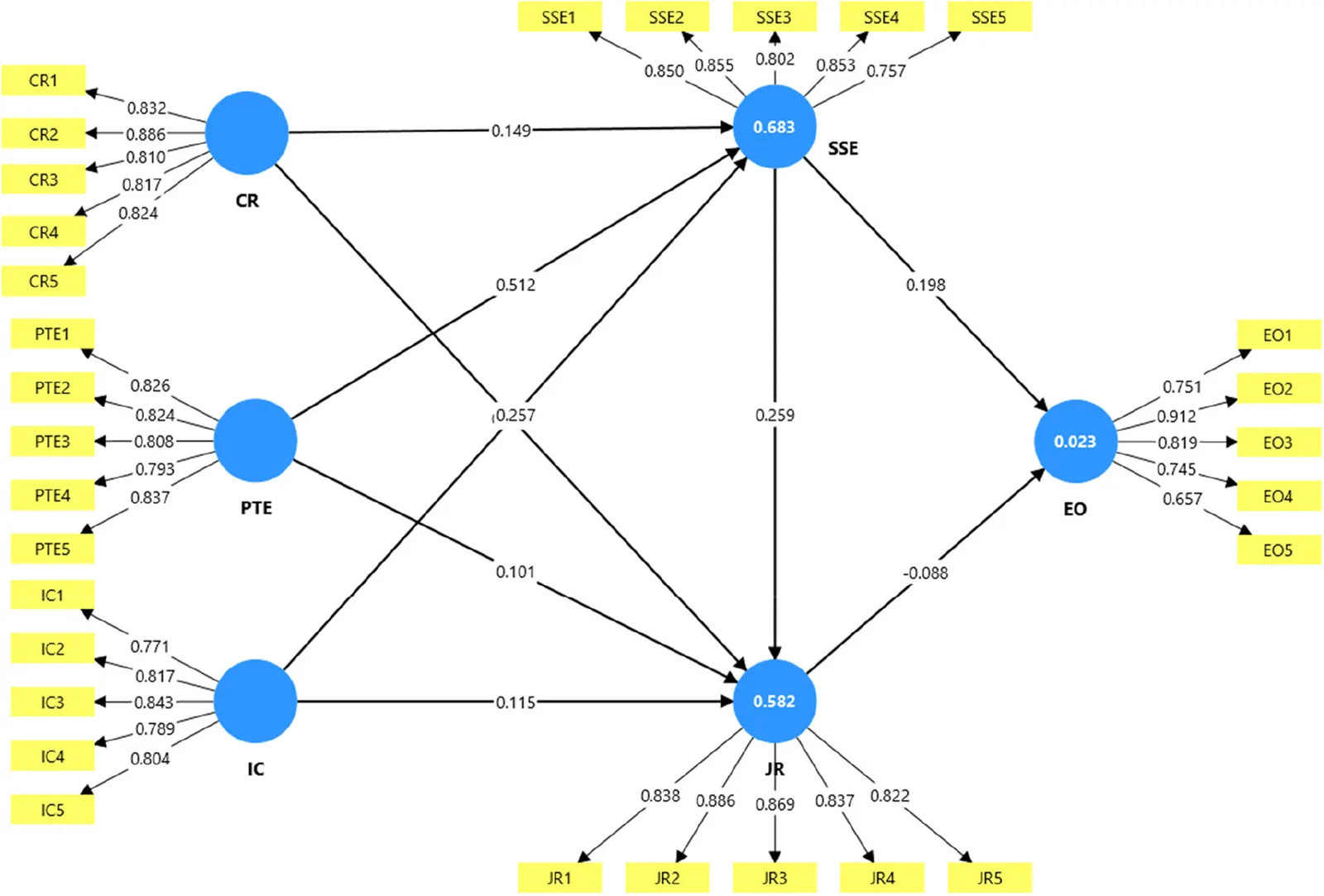
Measurement model with standardized factor loadings for all constructs

Indicator reliability was examined using standardized outer loadings, with values of 0.70 or higher considered to be indicative of adequate reliability. As reported in Table 2 and illustrated in Fig. 2, the majority of item loadings exceeded the recommended threshold, ranging from 0.657 to 0.912. Although one indicator showed a slightly lower loading (0.657), it was retained because it exceeded the minimum acceptable level of 0.60 and contributed to the overall content validity and construct reliability, consistent with recommended practices in PLS-SEM [16]. These results confirm that the indicators adequately represent the respective latent constructs.

**Table 2:** Results of the measurement model

Construct	Item*	Factor loading	Alpha (α)	(rho_a)	(rho_c)	AVE
Curriculum Relevance	CR1	0.832	0.890	0.892	0.920	0.696
	CR2	0.886				
	CR3	0.810				
	CR4	0.817				
	CR5	0.824				
Employment Outcomes	EO1	0.751	0.841	0.958	0.886	0.611
	EO2	0.912				
	EO3	0.819				
	EO4	0.745				
	EO5	0.657				
Instructor Competency	IC1	0.771	0.864	0.866	0.902	0.648
	IC2	0.817				
	IC3	0.843				
	IC4	0.789				
	IC5	0.804				
Job Readiness	JR1	0.838	0.904	0.906	0.929	0.724
	JR2	0.886				
	JR3	0.869				
	JR4	0.837				
	JR5	0.822				
Practical Training Exposure	PTE1	0.826	0.876	0.877	0.910	0.668
	PTE2	0.824				
	PTE3	0.808				
	PTE4	0.793				
	PTE5	0.837				
Student Self-efficacy	SSE1	0.850	0.881	0.884	0.914	0.679
	SSE2	0.855				
	SSE3	0.802				
	SSE4	0.853				
	SSE5	0.757				

Cronbach's alpha, Composite reliability (rho_a), Composite reliability (rho_c), and Average variance extracted (AVE)

Internal consistency reliability was assessed using Composite Reliability (CR) and Cronbach’s alpha, with values above 0.70 indicating satisfactory reliability. As shown in Table 2, the CR values ranged from 0.886 to 0.958, and the Cronbach’s alpha coefficients exceeded 0.84 for all constructs. These values fell within the recommended range, indicating a high level of internal consistency among the measurement items [17].

Convergent validity was evaluated using the Average Variance Extracted (AVE), with a threshold of 0.50 or higher, indicating that a construct explains more than half of the variance of its indicators. The AVE values for all constructs ranged from 0.611 to 0.724 (Table 2), exceeding the recommended threshold and confirming adequate convergent validity [11].

Discriminant validity was assessed using the heterotrait–monotrait (HTMT) ratio of correlations, applying both the conservative threshold of 0.85 and the more liberal threshold of 0.90 [17, 18]. As presented in Table 3, most HTMT values were below the conservative threshold of 0.85, indicating satisfactory discriminant validity for the constructs. However, the HTMT value between practical training exposure and student self-efficacy was 0.898, exceeding the conservative threshold but remaining below the more liberal threshold of 0.90. This suggests that the discriminant validity between these two constructs is acceptable but should be interpreted with caution. An elevated HTMT value indicates a potential conceptual overlap, which is theoretically plausible given the close relationship between experiential learning and self-efficacy within the Social Cognitive Theory. Furthermore, minor deviations from the conservative HTMT threshold are considered acceptable in exploratory and prediction-oriented PLS-SEM studies, particularly when the constructs are conceptually related [17, 18]. Therefore, while the results indicate some degree of construct proximity, they do not substantially undermine the overall discriminant validity of the measurement model.

**Table 3:** Discriminant validity using Heterotrait-Monotrait ratio (H.T.M.T)

	CR	EO	IC	JR	PTE	SSE
CR						
EO	0.126					
IC	0.700	0.163				
JR	0.787	0.060	0.668			
PTE	0.844	0.112	0.747	0.748		
SSE	0.778	0.147	0.781	0.762	0.898	

*CR* Curriculum Relevance, *EO* Employment Outcomes, *IC* Instructor Competency, *JR* Job Readiness, *PTE* Practical Training Exposure, *SSE* Student Self-efficacy

To further support discriminant validity, the Fornell–Larcker criterion was tested. As shown in Table 4, the square root of the AVE for each construct exceeded its correlations with the other constructs, indicating that each construct shares more variance with its indicators than with the other constructs [11]. This provides additional evidence of the distinctiveness of the constructs.

**Table 4:** Fornell-Larcker criterion

	CR	EO	IC	JR	PTE	SSE
CR	0.834					
EO	0.111	0.781				
IC	0.614	0.144	0.805			
JR	0.707	0.047	0.592	0.851		
PTE	0.747	0.104	0.650	0.667	0.818	
SSE	0.689	0.139	0.682	0.681	0.791	0.824

*CR* Curriculum Relevance, *EO* Employment Outcomes, *IC* Instructor Competency, *JR* Job Readiness, *PTE* Practical Training Exposure, *SSE* Student Self-efficacy

In addition, cross-loadings were assessed to ensure that each indicator loaded the highest on its associated construct compared to all other constructs. As presented in Table 5, all items demonstrated higher loadings on their respective constructs than on any other construct, confirming adequate discriminant validity at the indicator level and minimizing concerns of cross-loading or construct overlap [16].

**Table 5:** Cross loadings

	CR	EO	IC	JR	PTE	SSE
CR1	**0.832**	0.104	0.532	0.620	0.646	0.574
CR2	**0.886**	0.137	0.555	0.595	0.670	0.603
CR3	**0.810**	0.096	0.510	0.549	0.586	0.529
CR4	**0.817**	0.039	0.463	0.580	0.605	0.591
CR5	**0.824**	0.084	0.497	0.601	0.605	0.575
EO1	0.043	**0.751**	0.122	0.011	0.084	0.070
EO2	0.117	**0.912**	0.172	0.083	0.126	0.178
EO3	0.091	**0.819**	0.145	0.044	0.061	0.094
EO4	0.085	**0.745**	0.028	−0.007	0.070	0.077
EO5	0.076	**0.657**	0.052	0.012	0.026	0.064
IC1	0.453	0.121	**0.771**	0.434	0.506	0.539
IC2	0.485	0.157	**0.817**	0.516	0.523	0.525
IC3	0.489	0.107	**0.843**	0.527	0.531	0.575
IC4	0.530	0.139	**0.789**	0.442	0.521	0.554
IC5	0.514	0.056	**0.804**	0.458	0.538	0.552
JR1	0.610	0.057	0.492	**0.838**	0.554	0.565
JR2	0.636	0.011	0.522	**0.886**	0.565	0.619
JR3	0.620	0.062	0.516	**0.869**	0.582	0.586
JR4	0.575	0.056	0.489	**0.837**	0.550	0.552
JR5	0.563	0.015	0.497	**0.822**	0.586	0.573
PTE1	0.673	0.047	0.577	0.590	**0.826**	0.669
PTE2	0.622	0.064	0.545	0.579	**0.824**	0.640
PTE3	0.588	0.126	0.484	0.534	**0.808**	0.617
PTE4	0.558	0.085	0.506	0.482	**0.793**	0.620
PTE5	0.607	0.105	0.542	0.534	**0.837**	0.681
SSE1	0.562	0.163	0.548	0.595	0.640	**0.850**
SSE2	0.580	0.210	0.589	0.570	0.698	**0.855**
SSE3	0.595	0.033	0.559	0.573	0.643	**0.802**
SSE4	0.567	0.105	0.597	0.558	0.683	**0.853**
SSE5	0.537	0.047	0.512	0.508	0.588	**0.757**

Note. Boldface values represent the primary loading of each indicator on its theoretically assigned construct. They are used for identification purposes only and do not indicate statistical significance. Boldface values in Table 5 represent the primary loading of each indicator on its theoretically assigned construct and are used for identification purposes only; they do not indicate statistical significance

Overall, the measurement model demonstrated satisfactory reliability and validity, with acceptable indicator reliability, strong internal consistency, adequate convergent validity, and generally acceptable discriminant validity for all constructs. These results provide a robust foundation for subsequent structural analyses.

#### Structural model

**Fig. 3:**
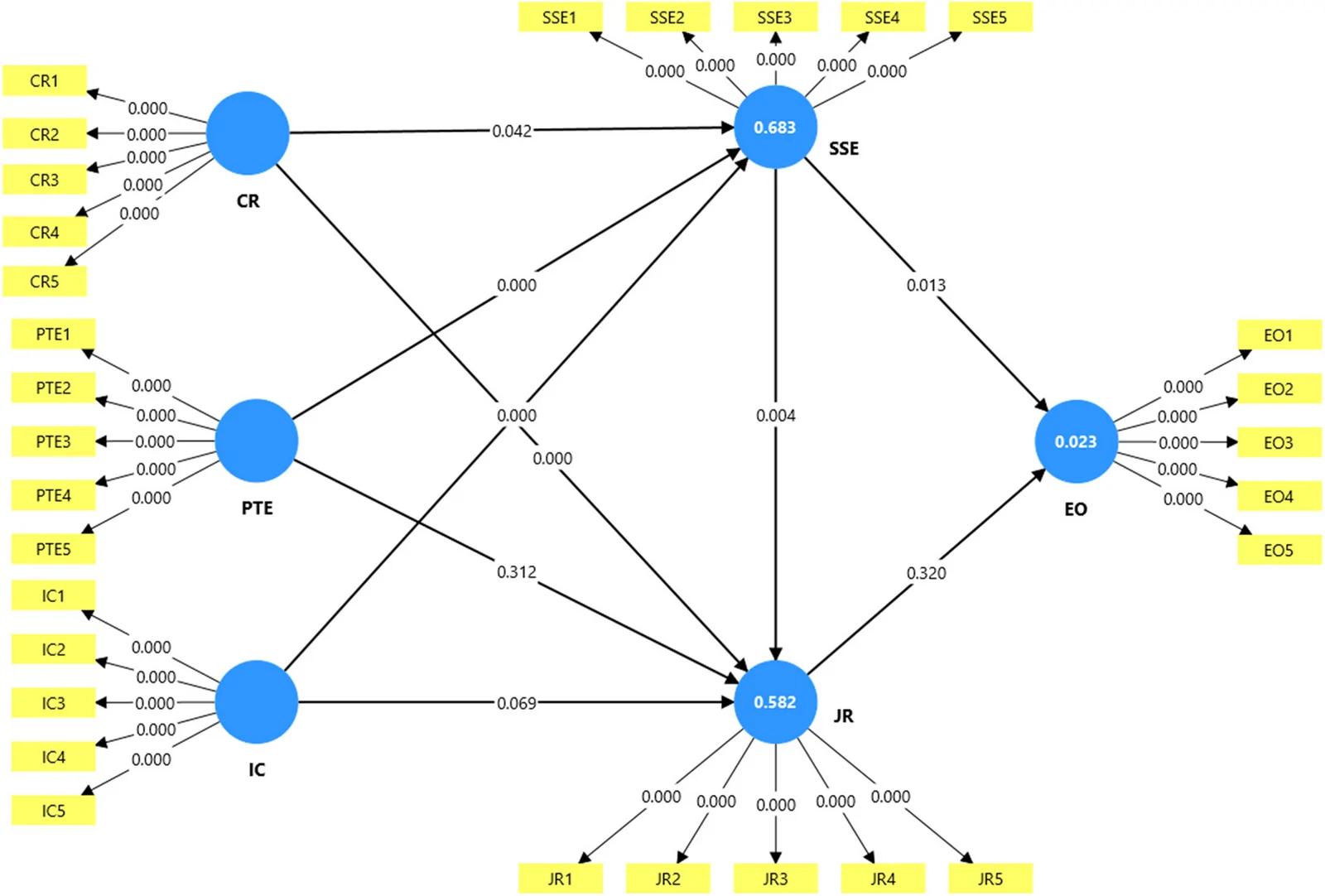
PLS-SEM structural model with standardized estimates and R^2^ values

The structural model was evaluated to examine the hypothesized relationships among curriculum relevance, practical training exposure, instructor competency, student self-efficacy, job readiness, and employment outcomes. Path coefficients (β), statistical significance, and explanatory power were assessed using bootstrapping with 5,000 resamples. The standardized structural relationships and coefficients of determination (R^2^) are illustrated in Fig. 3, and detailed numerical results are reported in Tables 6 and 7.

**Table 6:** Quality criteria

	f-square	VIF
CR—> JR	0.140	2.489
CR—> SSE	0.029	2.419
IC—> JR	0.015	2.060
IC—> SSE	0.113	1.851
JR—> EO	0.004	1.865
PTE—> JR	0.007	3.441
PTE—> SSE	0.316	2.614
SSE—> EO	0.022	1.865
SSE—> JR	0.051	3.153

**Table 7:** Hypothesis testing

Hypothesis	Structural Path	β (O)	Mean (M)	STDEV	t-value	*p*-value	Decision
H1	CR → SSE	0.149	0.150	0.073	2.031	0.042	Supported
H2	PTE → SSE	0.512	0.515	0.071	7.214	0.000	Supported
H3	IC → SSE	0.257	0.254	0.070	3.684	0.000	Supported
H4	CR → JR	0.382	0.389	0.092	4.163	0.000	Supported
H5	PTE → JR	0.101	0.107	0.100	1.012	0.312	Not Supported
H6	IC → JR	0.115	0.113	0.063	1.818	0.069	Not Supported
H7	SSE → JR	0.259	0.248	0.091	2.856	0.004	Supported
H8	JR → EO	− 0.088	− 0.092	0.088	0.995	0.320	Not Supported
H9	SSE → EO	0.198	0.212	0.080	2.484	0.013	Supported

As presented in Table 7 and visualized in Fig. 3, curriculum relevance had a significant positive effect on student self-efficacy (β = 0.149, t = 2.031, *p* = 0.042), and job readiness (β = 0.382, t = 4.163, *p *< 0.001). Practical training exposure strongly predicted student self-efficacy (β = 0.512, t = 7.214, *p* < 0.001), but its direct effect on job readiness was not significant (β = 0.101, t = 1.012, *p* = 0.312). Instructor competency showed a significant positive relationship with student self-efficacy (β = 0.257, t = 3.684, *p* < 0.001), whereas its direct relationship with job readiness was not significant (β = 0.115, t = 1.818, *p* = 0.069).

Student self-efficacy significantly and positively affected job readiness (β = 0.259, t = 2.856, *p* = 0.004) and employment outcomes (β = 0.198, t = 2.484, *p* = 0.013). In contrast, job readiness did not significantly predict employment outcomes (β = − 0.088, t = 0.995, *p* = 0.320). Overall, six of the nine hypothesized relationships were supported (Table 7).

The explanatory power of the structural model was assessed using the coefficients of determination (R^2^) for the endogenous constructs. As illustrated in Fig. 3, the model explained 68.3% of the variance in student self-efficacy (R^2^ = 0.683) and 58.2% of the variance in job readiness (R^2^ = 0.582), indicating a substantial predictive accuracy. In contrast, the model explained only 2.3% of the variance in employment outcomes (R^2^ = 0.023), indicating that the model has very limited explanatory power for employment outcomes and that additional contextual and labor market factors not included in this study are likely to play a substantial role in employment outcomes. Therefore, the findings related to employment outcomes should be interpreted cautiously and should not be taken as strong evidence that the model fully explains employability or actual labor market entry among medical laboratory graduates.

Effect sizes were evaluated using Cohen’s f^2^ to determine the relative contribution of each exogenous construct to the endogenous variables. As reported in Table 6, practical training exposure demonstrated a strong effect on student self-efficacy (f^2^ = 0.316), while curriculum relevance showed a moderate effect on job readiness (f^2^ = 0.140). Instructor competency exhibited a small effect on student self-efficacy (f^2^ = 0.113), while student self-efficacy showed small effects on job readiness (f^2^ = 0.051) and employment outcomes (f^2^ = 0.022).

Importantly, the effect size of job readiness on employment outcomes was extremely small (f^2^ = 0.004), indicating a practically negligible contribution despite its theoretical relevance. Similarly, practical training exposure (f^2^ = 0.007) and instructor competency (f^2^ = 0.015) showed minimal direct effects on job readiness. Overall, these results suggest that most structural relationships contribute incrementally, with only a few paths demonstrating substantive practical significance. Therefore, statistically significant relationships with small effect sizes, such as curriculum relevance to student self-efficacy, student self-efficacy to job readiness, and student self-efficacy to employment outcomes, should be interpreted as meaningful but modest effects rather than strong practical effects.

Multicollinearity was assessed using Variance Inflation Factor (VIF) values. All VIF values ranged from 1.851 to 3.441, remaining below the conservative threshold of 5.0, thereby confirming the absence of multicollinearity concerns and supporting the stability of the estimated path coefficients (Table 6).

#### Mediation analysis

Mediation analysis was conducted to examine the indirect effects of training quality factors on job readiness and employment outcomes through student self-efficacy and job readiness using bootstrapping procedures. As presented in Table 8, the results provide partial support for the proposed mediation hypotheses.

**Table 8:** Indirect effects

Hypothesis	Indirect Path	β (O)	Mean (M)	STDEV	t-value	*p*-value	Decision
H10	CR → SSE → JR	0.039	0.037	0.023	1.666	0.096	Not Supported
H11	PTE → SSE → JR	0.133	0.126	0.046	2.886	0.004	Supported
H12	IC → SSE → JR	0.067	0.065	0.034	1.960	0.050	Supported (marginal)
H13	CR → SSE → EO	0.029	0.032	0.021	1.418	0.156	Not Supported
H14	PTE → SSE → EO	0.102	0.109	0.043	2.347	0.019	Supported
H15	IC → SSE → EO	0.051	0.054	0.026	1.935	0.053	Supported (marginal)
H16	CR → JR → EO	−0.034	−0.035	0.034	0.974	0.330	Not Supported
H17	PTE → JR → EO	−0.009	−0.011	0.017	0.533	0.594	Not Supported
H18	IC → JR → EO	−0.010	−0.010	0.013	0.805	0.421	Not Supported
H19	SSE → JR → EO	−0.023	−0.024	0.026	0.867	0.386	Not Supported
H20	CR → SSE → JR → EO	−0.003	−0.004	0.005	0.701	0.484	Not Supported
H21	IC → SSE → JR → EO	−0.006	−0.006	0.008	0.763	0.446	Not Supported
H22	PTE → SSE → JR → EO	−0.012	−0.012	0.013	0.886	0.376	Not Supported

Specifically, student self-efficacy significantly mediated the relationship between practical training exposure and job readiness (β = 0.133, t = 2.886, *p* = 0.004), thus supporting H11. Similarly, student self-efficacy significantly mediated the relationship between practical training exposure and employment outcomes (β = 0.102, t = 2.347, *p* = 0.019), thus supporting H14. Additionally, the indirect effect of instructor competency on job readiness through student self-efficacy was marginally significant (β = 0.067, t = 1.960, *p* = 0.050), providing partial support for H12. Likewise, the mediating effect of student self-efficacy in the relationship between instructor competency and employment outcomes approached statistical significance (β = 0.051, t = 1.935, *p* = 0.053), indicating marginal support for H15.

In contrast, student self-efficacy did not significantly mediate the relationship between curriculum relevance and job readiness (β = 0.039, *p* = 0.096) or employment outcomes (β = 0.029, *p* = 0.156), leading to the rejection of H10 and H13.

Regarding mediation through job readiness, none of the indirect effects were statistically significant. Specifically, job readiness did not mediate the relationships between curriculum relevance, practical training exposure, or instructor competency and employment outcomes (H16–H18), nor did it mediate the relationship between student self-efficacy and employment outcomes (H19). All corresponding indirect paths were non-significant (*p* > 0.05).

Furthermore, the results did not support the proposed sequential mediation pathways involving student self-efficacy and job readiness. The indirect effects of curriculum relevance, instructor competency, and practical training exposure on employment outcomes through the sequential pathway (SSE → JR) were all non-significant (H20–H22), indicating that the hypothesized multistage mediation mechanism was not empirically supported in this study.

To clarify the theoretical meaning of the supported and unsupported hypotheses, Table 9 summarizes the main empirical patterns and their implications for Social Cognitive Theory, Social Cognitive Career Theory, and employability research in post-conflict health education contexts.

**Table 9:** Summary of supported theoretical implications and unsupported hypotheses

Result category	Supported/unsupported hypotheses	Main finding	Theoretical implication
Training quality and student self-efficacy	H1, H2, H3 supported	Curriculum relevance, practical training exposure, and instructor competency significantly predicted student self-efficacy	This supports Social Cognitive Theory by showing that relevant curricula, practical exposure, and instructor support strengthen students’ confidence in performing professional laboratory tasks
Training quality and job readiness	H4 supported; H5 and H6 not supported	Curriculum relevance significantly predicted job readiness, whereas practical training exposure and instructor competency did not directly predict job readiness	This suggests that curriculum alignment may contribute more directly to perceived readiness than practical exposure or instructor competency alone. Practical and instructional factors may influence readiness mainly through self-efficacy rather than direct pathways
Self-efficacy as a readiness mechanism	H7 supported	Student self-efficacy significantly predicted job readiness	This supports Social Cognitive Career Theory by confirming that psychological confidence is an important antecedent of professional readiness
Employment outcomes pathway	H9 supported; H8 not supported	Student self-efficacy significantly predicted employment outcomes, but job readiness did not	This indicates that employment outcomes in the study context may depend more on psychological confidence and broader labor market conditions than on perceived job readiness alone
Mediation through student self-efficacy	H11 and H14 supported; H12 and H15 marginally supported; H10 and H13 not supported	Student self-efficacy mediated the effects of practical training exposure on job readiness and employment outcomes, while instructor competency showed marginal indirect effects	This highlights self-efficacy as the strongest psychological bridge between practical training and employability-related outcomes
Mediation through job readiness	H16, H17, H18, H19 not supported	Job readiness did not mediate the relationship between training quality factors, student self-efficacy, and employment outcomes	This suggests that job readiness alone may be insufficient to explain employment outcomes in a fragile labor market where institutional and structural barriers influence workforce entry
Sequential mediation pathway	H20, H21, H22 not supported	The sequential pathway from training quality to employment outcomes through student self-efficacy and job readiness was not supported	This indicates that the transition from education to employment may not follow a simple linear process in post-conflict health systems. Future employability models should incorporate labor market, institutional, licensing, and employer-side variables

## Discussion

This study examined how curriculum relevance, practical training exposure, and instructor competency are associated with employability outcomes among medical laboratory graduates in post-conflict Somalia, with student self-efficacy and job readiness as mediating variables. In addition, mediation analysis provided further insight into the indirect pathways linking training quality factors to job readiness and employment outcomes. The findings demonstrate that curriculum relevance and practical training exposure significantly enhance student self-efficacy, while instructor competency also contributes positively to the development of self-efficacy. These results strongly align with Social Cognitive Theory, which emphasizes mastery experiences, feedback, and learning environments as primary sources of self-efficacy formation [2], and with Social Cognitive Career Theory, which positions self-efficacy as a central driver of career readiness and adaptive career behaviors [24]. From a Human Capital Theory perspective, the results indicate that investments in relevant curricula and experiential learning may strengthen productive capabilities that translate into employability-relevant competencies [4]. Rather than indicating direct employability enhancement, these findings suggest that training quality first operates through psychological readiness mechanisms. In the Somali post-conflict context, curriculum relevance, practical exposure, and instructor support may help students develop confidence in their laboratory abilities, but this confidence may not automatically translate into employment because labor market entry is also shaped by institutional and structural constraints.

The positive relationship between curriculum relevance and self-efficacy and job readiness is consistent with prior evidence from graduate employability research, which emphasizes the alignment of academic content with labor market demands as a critical determinant of graduate confidence and employability outcomes [8, 33]. Similarly, the strong effect of practical training exposure on self-efficacy supports the findings from health professions education, showing that experiential learning and clinical placements enhance professional competence and confidence [10, 12]. The significant influence of instructor competency on self-efficacy also aligns with evidence that effective supervision and instructional quality shape learner confidence and professional identity development [12]. Importantly, mediation analysis revealed that student self-efficacy significantly mediated the relationship between practical training exposure and both job readiness and employment outcomes, highlighting its central role as a psychological mechanism through which training quality is translated into employability-related outcomes. This theoretical interpretation strengthens the role of self-efficacy as more than a statistical mediator; it suggests that students’ confidence may function as the psychological bridge between educational experiences and early employability perceptions. In medical laboratory education, hands-on training and instructor guidance may therefore be most valuable when they develop students’ belief that they can perform diagnostic tasks, adapt to clinical environments, and engage with workplace expectations.

However, the nonsignificant direct effects of practical training exposure and instructor competency on job readiness diverge from some studies conducted in stable higher education systems, where training intensity directly predicts readiness and employability [8]. This divergence may reflect structural constraints in post-conflict contexts, including limited clinical infrastructure, inconsistent supervision quality, and constrained labor market absorption, which may weaken the direct translation of training inputs into perceived readiness. The significant pathway from self-efficacy to job readiness reinforces SCCT propositions that psychological confidence precedes behavioral readiness and adaptive performance [24]. Nevertheless, the mediation results indicate that job readiness does not function as a significant mediator between training quality factors and employment outcomes, as none of the indirect effects of job readiness were statistically significant. Conceptually, job readiness and employment outcomes represent related but distinct constructs in this study. Job readiness reflects graduates’ perceived technical, behavioral, and professional preparedness for entering the workplace, whereas employment outcomes capture broader employability-related indicators, including perceived employability, current employment status, and alignment with current or expected job roles. Therefore, a graduate may feel professionally ready but may still not obtain employment or role alignment because actual employment outcomes depend on external labor market and institutional conditions. This finding differs from prior employability and SEM-based studies that position job readiness as a stronger proximal predictor of employment outcomes. In the present post-conflict context, job readiness may not translate directly into employment because employment opportunities are shaped not only by graduate preparedness but also by institutional instability, limited health-sector vacancies, weak transition-to-employment pathways, and constrained labor market absorption. The lack of a significant direct effect of job readiness on employment outcomes further suggests that macro-level labor market dynamics, institutional instability, and limited employment opportunities may mediate employment attainment in fragile settings, consistent with workforce studies in low-resource health systems [5, 15, 25, 31, 32].

However, the model’s explanatory power for employment outcomes was limited (R^2^ = 0.023), indicating that the included variables accounted for only a small proportion of the variance in employability. This very low R^2^ further supports the interpretation that employment outcomes among medical laboratory graduates in Somalia cannot be explained adequately by individual-level readiness variables alone. Instead, the result suggests that broader contextual factors, such as labor market saturation, institutional hiring practices, professional licensing barriers, and health-sector workforce absorption, may play a more decisive role in determining actual employment outcomes. Accordingly, the discussion of employment outcomes should be understood as an interpretation of a weakly explained outcome rather than evidence that training quality or job readiness strongly predicts employment. Therefore, the findings related to employment outcomes should be interpreted with caution and viewed as providing partial and context-specific insights rather than a comprehensive explanation of employability.

The broad operationalization of employment outcomes may also partly explain the limited explanatory power of the model. Because the construct combines subjective perceptions of employability with objective workforce-position indicators, it captures multiple dimensions of early career development that may be influenced by different educational, psychological, and labor market factors. This interpretation reinforces the need to distinguish between graduate preparedness for employment and actual employment attainment in post-conflict labor markets.

Theoretically, this study extends employability research by empirically validating a multistage mediation model linking educational quality to employment-related outcomes through psychological and professional readiness mechanisms. The results reinforce the explanatory power of SCT and SCCT in fragile contexts while demonstrating the applicability of Human Capital Theory beyond purely economic settings. Methodologically, this study contributes to the limited body of SEM-based employability research in low-income and post-conflict health education systems by offering a robust structural modeling approach that integrates educational quality, psychological constructs, and labor market outcomes. However, the lack of support for sequential mediation pathways suggests that the transition from self-efficacy to employment outcomes may not operate through a fully sequential process involving job readiness in this context, indicating the need for refinement of theoretical models in fragile and resource-constrained environments.

Overall, the study demonstrates that employability development in post-conflict health education systems is more strongly associated with psychological readiness mechanisms shaped by training quality than with direct instructional inputs alone. Specifically, the findings underscore that student self-efficacy plays a more critical mediating role than job readiness in linking training quality to employment outcomes. Transparent reporting, rigorous SEM validation, and theory-driven model specifications strengthen the credibility and policy relevance of the findings.

The findings of this study offer several important implications for educational practice, health workforce policy, and future research in post-conflict contexts.

From a practical perspective, the results highlight the importance of strengthening curriculum relevance and structured practical training to enhance students’ self-efficacy and job readiness. Medical laboratory education programs should prioritize competency-based curricula that closely align with clinical laboratory standards and evolving diagnostic technologies. In addition, expanding access to hands-on training through simulation-based laboratories and structured clinical placements may provide students with meaningful opportunities to develop their technical competence and professional confidence. Given the resource constraints in post-conflict settings, low-cost simulation approaches and partnerships with public and private healthcare facilities may represent feasible strategies for improving experiential learning. These educational strategies should be interpreted primarily as ways to strengthen readiness and professional confidence, rather than as sufficient solutions for employment outcomes.

From a policy perspective, the findings suggest that improving employability outcomes requires coordinated efforts beyond the educational institutions. Policymakers and regulatory bodies should support the development of stronger university–clinical partnerships, standardized training frameworks, and accreditation mechanisms to ensure the consistent quality of laboratory education. Investment in faculty development is also critical, as instructor competency plays a significant role in shaping students’ self-efficacy. In addition, policies aimed at strengthening health workforce absorption, such as structured internship programs, transition-to-employment schemes, and incentives for healthcare facilities to recruit new graduates, may help address the gap between training and employment in fragile health systems. Importantly, the very low explained variance in employment outcomes suggests that employability in post-conflict settings may be associated with broader structural and labor market conditions. Therefore, policy interventions must consider systemic challenges, including limited job availability, institutional instability, and resource constraints within the health sector.

From a research perspective, this study underscores the need for more comprehensive and context-sensitive employability models in low-resource and post-conflict environments. Future studies should incorporate macro-level variables, such as labor market dynamics, institutional capacity, professional licensing systems, institutional hiring practices, and regulatory frameworks, to improve the explanatory power. Longitudinal research designs are recommended to capture transitions from education to employment over time. Future research should also consider employer perspectives, objective competency assessments, gender-based analyses, and subgroup comparisons to better understand the potential differences in employability pathways across demographic groups.

## Conclusion

This study provides empirical evidence on how training quality factors are associated with employability outcomes among medical laboratory graduates in post-conflict Somalia, using a theory-driven Structural Equation Modeling framework. The findings demonstrate that curriculum relevance, practical training exposure, and instructor competency significantly enhance student self-efficacy, which, in turn, is associated with higher levels of job readiness and contributes to employment outcomes. However, the findings related to employment outcomes should be interpreted with caution, as the structural model explained only a small proportion of the variance in employment outcomes (R^2^ = 0.023). This suggests that training quality, self-efficacy, and job readiness alone are insufficient to explain actual employment outcomes, and that broader labor market, institutional, and socioeconomic factors may play a more substantial role.

The results extend the SCT and Social Cognitive Career Theory by supporting multistage pathways linking educational quality to employability-related outcomes in fragile health systems. The integration of Human Capital Theory and graduate employability models further suggests that training quality may contribute to labor market outcomes primarily through capability development and confidence formation rather than direct instructional effects alone. These theoretical implications should be interpreted within the context and methodological boundaries of the study.

From a policy and practice perspective, the findings highlight the importance of curriculum alignment, experiential learning, and instructor quality in strengthening students’ psychological readiness. While the findings support the importance of curriculum relevance, practical training exposure, instructor competency, and student self-efficacy in enhancing graduate preparedness, the model’s very low explanatory power for employment outcomes (R^2^ = 0.023) suggests that employability is influenced by factors beyond the educational environment. In post-conflict Somalia, labor market conditions, health sector recruitment capacity, professional licensing systems, institutional hiring practices, and broader socioeconomic constraints may play a more substantial role in determining employment outcomes than educational factors alone. Therefore, efforts to improve graduate employability should extend beyond curriculum reform and training quality enhancement to include stronger university-employer partnerships, workforce planning mechanisms, health sector job creation initiatives, and policies that facilitate the transition of graduates into the labor market.

While the findings are context-specific, they provide indicative insights for similar fragile and post-conflict education systems seeking to strengthen their health workforce capacity. However, generalization beyond comparable contexts should be approached with caution.

Overall, this study contributes to an improved understanding of employability development in post-conflict health education systems while acknowledging the complexity of factors influencing labor market outcomes. Importantly, the model demonstrated very limited predictive power for employment outcomes (R^2^ = 0.023), indicating that employability in post-conflict settings is largely shaped by external structural, institutional, and labor market factors beyond this study’s scope.

### Limitations and future research

This study had several methodological limitations. First, the cross-sectional design limits causal inference and does not capture temporal dynamics in the relationship between training quality, psychological readiness, and employment outcomes. Therefore, the findings should be interpreted as associative rather than causal. Future research employing longitudinal designs would provide stronger evidence of the transition from education to employment over time.

Second, the use of purposive non-probability sampling and focus on institutions in Mogadishu may restrict the generalizability of the findings to other regions or educational contexts. Given the context-specific nature of post-conflict health systems, caution is required when extending these results beyond similar institutional and geographical settings. As participants were selected based on predefined inclusion criteria rather than random sampling, the sample may not fully represent all medical laboratory students and graduates in Somalia. Therefore, the findings should be interpreted as context-specific evidence from Mogadishu, rather than as nationally representative conclusions. Future studies should consider probability-based or multi-site sampling approaches across different regions of Somalia to strengthen the external validity and improve the representativeness of the findings.

Third, the gender imbalance represents an important limitation of this study. The sample exhibited a gender imbalance (68.4% male), which may affect representativeness and limit the applicability of the findings across sex groups. Although this distribution may reflect enrollment and participation patterns within medical laboratory education in the study context, it may limit the extent to which the findings represent the experiences and perceptions of female students and graduates in general. Differences in educational access, social expectations, and labor market opportunities between male and female graduates may influence their employability pathways. Future studies should consider gender-balanced samples or subgroup analyses to examine whether the relationships among training quality, student self-efficacy, job readiness, and employment outcomes differ by sex.

Fourth, all variables were measured using self-reported data collected at a single point in time, which may have introduced common method and social desirability biases. Although statistical procedures such as full collinearity assessment were applied, the potential influence of common method variance cannot be entirely ruled out in this study. Additionally, because respondents provided information on training quality, psychological readiness, job readiness, and employment outcomes using the same questionnaire, shared method effects may have inflated or weakened some observed relationships. Although procedural measures, including anonymity, voluntary participation, and clear instructions, were used to reduce response bias, future studies should combine self-reported data with objective indicators, employer assessments, clinical performance records, or longitudinal follow-up data to strengthen the validity and reduce common method concerns.

Fifth, employment outcomes were operationalized as perceived employability and alignment with current roles, rather than objective employment indicators. This may limit the ability to fully capture actual labor market outcomes, particularly in contexts where a large proportion of respondents were engaged in internships or volunteer positions. Because the employment outcomes construct combines perceived employability, employment status, and role alignment, it may reflect a broader employability-related outcome rather than actual employment attainment alone. Future studies should more clearly distinguish between subjective employability perceptions and objective employment indicators, such as job placement, contract status, salary level, and time to first employment.

Finally, the structural model explained only a small proportion of the variance in employment outcomes (R^2^ = 0.023), indicating that important external factors, such as labor market conditions, institutional hiring practices, professional licensing systems, and broader socioeconomic constraints, were not captured in the model. Future research should incorporate these contextual and macro-level variables to improve the explanatory power and provide a more comprehensive understanding of employability in post-conflict settings. This low explanatory power also suggests that the model is more adequate for explaining psychological and job readiness than for fully explaining employment outcomes. Therefore, the employment outcomes model should be interpreted cautiously as a partial and exploratory component of the broader employability framework, rather than as a complete predictive model of actual labor market entry.

## Data Availability

The datasets generated and analyzed during the current study are not publicly available due to ethical and confidentiality considerations involving human participants but are available from the corresponding author upon reasonable request, subject to approval by the relevant ethics committee.
